# Cell Cycle-Regulated Protein Abundance Changes in Synchronously Proliferating HeLa Cells Include Regulation of Pre-mRNA Splicing Proteins

**DOI:** 10.1371/journal.pone.0058456

**Published:** 2013-03-08

**Authors:** Karen R. Lane, Yanbao Yu, Patrick E. Lackey, Xian Chen, William F. Marzluff, Jeanette Gowen Cook

**Affiliations:** 1 Curriculum in Genetics and Molecular Biology, University of North Carolina at Chapel Hill, Chapel Hill, North Carolina, United States of America; 2 Department of Biochemistry and Biophysics, University of North Carolina at Chapel Hill, Chapel Hill, North Carolina, United States of America; 3 Lineberger Comprehensive Cancer Center, University of North Carolina at Chapel Hill, Chapel Hill, North Carolina, United States of America; 4 Program in Molecular Biology and Biotechnology, University of North Carolina at Chapel Hill, Chapel Hill, North Carolina, United States of America; Instituto Butantan, Laboratório Especial de Toxinologia Aplicada, Brazil

## Abstract

Cell proliferation involves dramatic changes in DNA metabolism and cell division, and control of DNA replication, mitosis, and cytokinesis have received the greatest attention in the cell cycle field. To catalogue a wider range of cell cycle-regulated processes, we employed quantitative proteomics of synchronized HeLa cells. We quantified changes in protein abundance as cells actively progress from G1 to S phase and from S to G2 phase. We also describe a cohort of proteins whose abundance changes in response to pharmacological inhibition of the proteasome. Our analysis reveals not only the expected changes in proteins required for DNA replication and mitosis but also cell cycle-associated changes in proteins required for biological processes not known to be cell-cycle regulated. For example, many pre-mRNA alternative splicing proteins are down-regulated in S phase. Comparison of this dataset to several other proteomic datasets sheds light on global mechanisms of cell cycle phase transitions and underscores the importance of both phosphorylation and ubiquitination in cell cycle changes.

## Introduction

The cell cycle is highly regulated to ensure accurate duplication and segregation of chromosomes. Perturbations in cell cycle control can result in genome instability, cell death, and oncogenesis [1], [2], [3], [4]. Critical transition points in the cell cycle reflect “points of no return” that are difficult or impossible to reverse. For example, the G1 to S phase transition, marked by the onset of DNA replication, is an essentially irreversible step, as is mitosis. For this reason, the major cell cycle transitions into and out of S phase and mitosis are under particularly complex and robust control. The mechanisms that govern such cell cycle transitions include changes in protein abundance that are driven by combinations of regulated gene expression and protein stability control (reviewed in ref. [5]). Though decades of genetic and biochemical studies have given great insight into such mechanisms, much remains to be learned about the overall impact of cell cycle transitions on intracellular physiology.

To date, cell cycle studies have focused primarily on the regulation of DNA replication (S phase), chromosome segregation (M phase), and cytokinesis. A few recent unbiased analyses of cell cycle-associated changes in human mRNA abundance suggest that other biological processes are also cell cycle-regulated [6], [7]. Nevertheless, the full spectrum of cellular changes at the major cell cycle transitions is still unknown. In particular, the mRNA changes during the cell cycle in continuously growing cells are unlikely to reflect the rapid changes in concentrations of critical proteins. A 2010 study by Olsen *et al.* analyzed both changes in protein abundance and phosphorylation events in the human cell cycle, focusing primarily on changes in mitosis [8]. In this current study, we investigated protein abundance changes associated with S phase relative to both G1 and G2 in highly synchronous HeLa cells (human cervical epithelial carcinoma). In parallel, we have catalogued changes in the proteome in response to inhibition of ubiquitin-mediated degradation in synchronous cells. In addition to finding some of the previously-described changes related to DNA metabolism and mitosis, we also uncovered changes in many proteins involved in alternative pre-mRNA splicing.

## Materials and Methods

### Cell Culture and Synchronization

HeLa cells were originally obtained from ATCC and were cultured in three different media. “Light” cells were grown in depleted Dulbecco’s Modified Eagle Medium (DMEM; UCSF Cell Culture Facility, CCFDA003-102I3C) reconstituted with 145 mg/L L-lysine (UCSF Cell Culture Facility, CCFGA002-102M04) and 84 mg/L L-arginine (UCSF Cell Culture Facility, CCFGA002-102J1X). “Medium” cells were grown in depleted DMEM reconstituted with 798 mM L-lysine (^4,4,5,5^D_4_, DLM-2640) and 398 mM L-arginine (^13^C_6_, CLM-2265). “Heavy” cells were grown in depleted DMEM reconstituted with 798 mM L-lysine (^13^C_6_; ^15^N_2_, CNLM-291) and 398 mM L-arginine (^13^C_6_; ^15^N_4_, CNLM-539). All three media were supplemented to 10% dialyzed fetal bovine serum (dFBS; Gibco, 26400-044) and 2 mM L-glutamine (UCSF Cell Culture Facility, CCFGB002-101J04. All modified isotopes were purchased from Cambridge Isotope Laboratories, Inc. (Andover, MA). All HeLa cell cultures were grown in the SILAC media for a minimum of 5 passages to ensure that the amino acids had been fully incorporated. Labeling efficiency was checked by examination of the tubulin and actin proteins using LC-MS/MS (details of sample preparation and analysis follow). T98G cells were originally obtained from ATCC and were cultured in DMEM (Sigma Aldrich, D5648) supplemented with 10% FBS (Sigma Aldrich, F2442) and 2 mM L-glutamine (Gibco, 25030-081). Cells were synchronized by serum starvation for 72 hr and stimulated with a final concentration of 10% FBS [9].

To determine the protein changes between G1 and S phase, simultaneously cultured biological replicates of HeLa cells were subjected to double-thymidine synchronization as previously described in ref. [7] with minor modifications. Ten hours after release from the second thymidine block, the medium was removed, and a mitotic shake-off was performed. Mitotic cells were replated and collected at 3 hr (G1 sample) and 10 hr (S sample). To capture proteins degraded after S phase onset, one separately-labeled culture was treated with 20 µM MG132 (Sigma Aldrich, C2211) for 2 hr prior to harvest (8 hrs after shakeoff). To determine the protein changes between S and G2 phase, simultaneously cultured biological replicates were harvested 3 hr following release from the second thymidine treatment (S sample) and 8 hr after release (G2 sample); one separately-labeled culture received 20 µM MG132 2 hr prior to harvesting in G2. Cells were harvested by trypsinization, collected by centrifugation, and cell pellets were stored at −80°C prior to the preparation of cell lysates. A small fraction of cells was fixed with ethanol, stained with propidium iodide, and analyzed by flow cytometry to confirm cell cycle phase.

### Cell Lysis and Sample Processing

Frozen cell pellets were lysed in 50 µL high salt lysis buffer (10 mM HEPES-KOH, pH 7.5 (H4034), 350 mM KCl (P9541), 3 mM MgCl_2_ (M8266), 1% Triton-X100 (T9284-100 mL), 1 mM EDTA, pH 8.0 (Fisher Scientific, S311-500)) and incubated on ice for 10 min. Lysis buffers were supplemented with 1 mM DTT (D0632-5G), 0.1 mM AEBSF (Roche, 11585916001), 0.5 mM NaOV_4_ (S6508-50G), 2 mM β-glycerolphosphate (G6376-25G), 2 mM NaF (201154-100G), 200 nM trichostatin A (T8552), 2.5 mM sodium butyrate (303410), and 1 µg/mL each of aprotinin (A1153), leupeptin (L2884), and pepstatin A (P5318). Unless otherwise indicated, all chemicals were purchased from Sigma Aldrich. Lysates were cleared by centrifugation for 2 min at 4°C; the supernatant was transferred to a new tube and cleared by centrifugation at full speed for 15 min at 4°C. Protein concentrations were determined according to Bradford assay instructions (Biorad, 500-0006). Samples were mixed 1∶1∶1 (70 µg each) and subjected to SDS-PAGE on a 15% polyacrylamide gel. The gel was stained with Coomassie blue (Amresco, M140-10G), and sample lanes were continuously excised into 25 slices. The following steps, including destaining, dehydration, reduction and alkylation, and overnight in-gel trypsin digestion, were performed following a standard protocol [10].

### Desalting and LC-MS/MS

After digestion, the peptides were extracted using C18 ziptips (Millipore, ZTC18S096), lyophilized, and resuspended in buffer A (0.1% formic acid in H_2_O) prior to LC separation. MS analyses were performed on an LTQ Orbitrap Velos (Thermo Scientific, Bremen, Germany) coupled with a nanoLC-Ultra system (Eksigent, Dublin, CA). Samples (5 µL) were loaded onto an IntegraFrit column (C18, 75 µm × 15 cm, 300 Å, 5 µm, New Objective, MA). The peptides were eluted at a flow rate of 200 nl/min with a linear gradient from 2% to 40% buffer B (0.1% formic acid in acetonitrile) over the course of 110 min, followed by 80% buffer B for another 10 min. At the end of the gradient, the column was equilibrated for 10 min with 2% buffer B before starting another LC/MS run. The mass spectrometer was programmed to acquire spectra in a data-dependent and positive ion mode at a spray voltage of 2.1 kV using the XCalibur software (version 2.1, Thermo Scientific). Survey scans were performed in the Orbitrap analyzer at a resolution of 15,000 over a mass range between m/z 300-2,000. For each cycle, the top five most intense ions were subjected to CID fragmentation in the LTQ with normalized collision energy at 35% and activation Q 0.25; dynamic exclusion was enabled. Selected ions were repeated once and then excluded from further analysis for 45 sec. Unassigned ions or those with a charge of 1+ were rejected. Maximum ion accumulation times were 200 ms for each full MS scan and 100 ms for MS/MS scans. One microscan was acquired for each MS and MS/MS scan. The mass spectrometry data from this publication have been submitted to the Proteome Commons Tranche (www.proteomecommons.org). The data from the G1 to S dataset can be found using the following hash code: ytUg3dJ7npt665b/ZRSADaIKbwhAbVLfVjOiV1qw0zUjr1f7rr+cJk6txiV+2CDE3cQEnKErNJ/mV6edECVH1yf4r70AAAAAAAAM5Q =  = . The data from the S to G2 dataset can be found using the following hash code: Pfr5X84wSDM2MuckUXaXkFAqfoq2r94aKYgVm7NCTmz4L/pd5OpHEfoz3CxrMJfnZe86hl8j2lJMDVZjSUkc1Du8hcQAAAAAAAAOuQ =  = .

### Database Search

The raw files were processed using the MaxQuant software suite (version 1.2.0.34) [11]. The MS/MS spectra were used to interrogate the UniProt human database (release date of November 30, 2010. 20248 entries) using the Andromeda search engine [12] with the precursor and fragment mass tolerances set to 6 ppm and 0.5 Da, respectively. Up to two missed cleavage sites were allowed per peptide. Methionine oxidation and protein N-terminal acetylation were chosen as variable modifications, and cysteine carabamidomethlyation was set as a fixed modification for database searching. Only peptides with a minimum length of 6 amino acids were considered for identification. Both peptide and protein identifications were filtered to a maximum 1% false discovery rate. Proteins identified from only a single peptide were manually checked by direct visualization of the spectra and quantified using the XCalibur software. Finally, the lists of identified proteins were filtered to eliminate reverse hits and known contaminants.

As a complement to MaxQuant the Proteome Discoverer software (version 1.3, Thermo Scientific), configured with an in-house Mascot server (v2.3, Matrix Science), was also used to search the same set of MS/MS data. A built-in workflow and a “Quantification” module were used for protein identification and quantitation. All the search parameters were the same as the MaxQuant search, but were filtered at a false discovery rate of 5% to quantify a similar number of proteins as had been identified with MaxQuant. Both search strategies generated overlapping protein lists (77%). Once results were gathered from both programs, the results were combined. When proteins were identified by both programs, the quantification calculated by the MaxQuant software was reported. If the ratios were such that one program defined a protein as changed whereas the second program did not, the ratios were manually calculated through integration of the peak areas using the XCalibur software.

Proteins were divided into subsets based on their SILAC ratios using a 1.5-fold change as the cutoff threshold. That is, a ratio of 1.5 or higher was scored as an increase whereas a ratio of 0.666 or less was scored as a decrease; ratios that fell between these values were reported as no change. These ratios, as well as the log_2_ transformations, are reported in Tables S1 and S2.

### Dataset Comparison and GO Term Analysis

The log_2_ transformed data from Whitfield et al. (2002) was downloaded from www.cyclebase.org. Based on the calculated p-value of periodicity, mRNA data were separated according to mRNA peak time [13], [14]. These lists were compared to our lists of increased and decreased proteins, and p-values were calculated using Fisher’s exact test; a p-value less than 0.01 was considered significant. The same strategy was applied to comparisons to the ubiquitome [15], a published ATM/ATR substrate list [16], a published phosphoproteome [8], a Cyclin A/Cdk2 substrate list [17], and a dataset that determined the subcellular localization of proteins [18]. GO term analysis was performed using the DAVID search engine [19], [20]. Analysis was performed on the individual lists, and the reported p-value was calculated using a modified Fisher’s exact test. When GO terms overlapped, terms were collapsed to the highest level (i.e., RNA splicing was collapsed into RNA processing).

### Immunoblot Validation

Samples were subjected to SDS-PAGE on a 12% polyacrylamide gel and transferred to PVDF (Thermo Scientific, 88518). Blots were probed with the following antibodies: anti-Cyclin B1 (V152, Thermo Scientific, MA1-46103), anti-Cyclin A (C-19, Santa Cruz Biotechnology, sc-596), anti-Cdc6 (D-1, Santa Cruz Biotechnology, sc-13136), anti-Cdt1 [21], anti-Geminin (FL-209, Santa Cruz Biotechnology, sc-13015), anti-SLBP [22], anti-α-tubulin (DM1A, Sigma Aldrich, 9026), anti-RRM2 (Aviva Systems Biology, ARP46031), anti-MARCKSL1 (Aviva Systems Biology, ARP64193), anti-Palmdephin (Aviva Systems Biology, ARP66420), anti-Prelamin A/C (N-18, Santa Cruz Biotechnology, sc-6215), anti-Tropomodulin-3 [23], anti-MCM2 (46/BM28, BD Pharmingen, 610700), anti-Rbmx/hnRNPG (Aviva Systems Biology, ARP61802), anti-hnRNPA1 (K350, Cell Signaling, 4296), anti-hnRNPA3 (Y25, Santa Cruz Biotechnology, sc-133665), anti-hnRNPD0 (T10, Santa Cruz Biotechnology, sc-22368), anti-hnRNPL (Sigma Aldrich, SAB1405954), and anti-β-actin (N-21, Santa Cruz Biotechnology, sc-130656). All HRP-conjugated secondary antibodies were purchased from Jackson Immunoresearch (DαR 711-035-152, GαM 115-035-146, BαG 805-035-180). Proteins were visualized following incubation with ECL prime reagent (Amersham, RPN2232).

## Results

### Synchronous HeLa Cells Progressing through the G1/S and S/G2 Transitions

We sought to investigate the proteome changes between G1 and S phase and between S and G2 phase. Our goal was to achieve very tight cell cycle synchrony while simultaneously avoiding strong checkpoint effects that could be induced in chemically-arrested cells. To facilitate accurate quantification of peptides by mass spectrometry, we labeled cultures for more than 5 cell divisions with three different stable isotope mixtures of lysine and arginine (i.e. amino acid-coded mass tagging/AACT or stable isotope labeling with amino acids in culture/SILAC) prior to synchronization [24], [25], [26].

**Figure 1:**
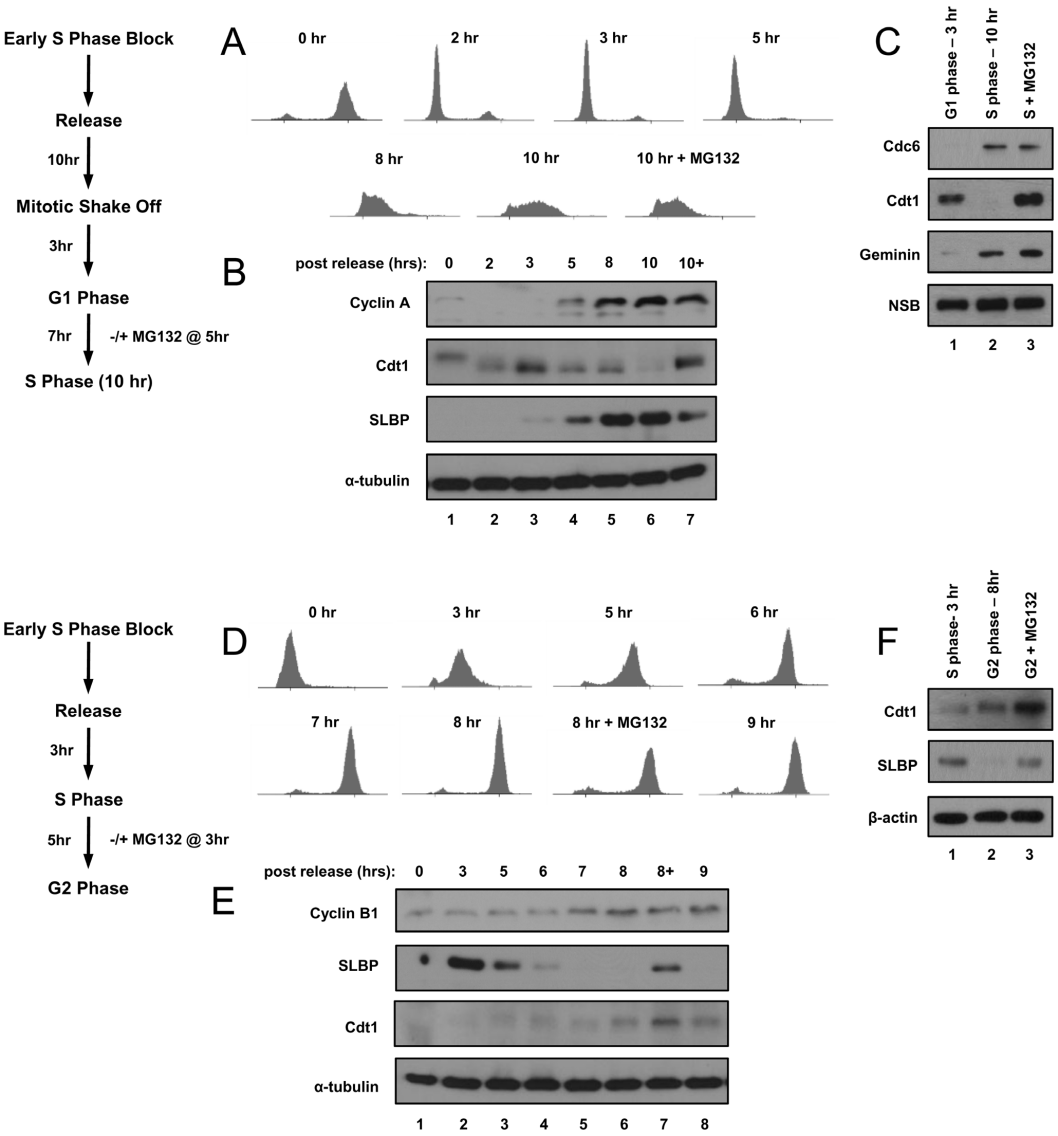
HeLa cell synchronization. A) Cells were synchronized by a modified double-thymidine block then released by re-plating and harvested at the indicated time points; a late G1 phase culture was treated with MG132 two hrs prior to harvest in early S phase. Synchrony was determined by flow cytometric analysis of DNA content. B) Immunoblot analysis of endogenous Cyclin A, Cdt1, SLBP, and tubulin proteins in whole cell lysates from portions of the same cells used in A. C) Cells were metabolically labeled with stable isotopes and then synchronized in G1 (3 hrs after mitosis, normal/“light” isotopes) and early-S phase (10 hrs after mitosis, labeled with intermediate or “medium” isotopes) as in A and B. Cells labeled with the heaviest isotopes were treated with MG132 two hrs prior to harvest in early S phase. Immunoblot analysis of endogenous Cdc6, Cdt1, and geminin in whole cell lysates used for subsequent mass spectrometric tests. A non-specific band (NSB) serves as a loading control. D) Cells were synchronized by double-thymidine block, released into S phase, and harvested at the indicated timepoints. Synchrony was determined by flow cytometric analysis of DNA content. E) Immunoblot analysis of endogenous Cyclin B, SLBP and Cdt1 in whole cell lysates from portions of the same cells used in D. F) Cells were metabolically labeled with stable isotopes and synchronized in S phase (light isotopes) or G2 phase (medium isotopes) as in D and E. A culture labeled with heavy isotopes was treated with MG132 in late S phase for two hrs prior to harvest in G2. Immunoblot analysis of endogenous Cdt1 and SLBP in whole cell lysates used for subsequent mass spectrometric analysis; β-actin serves as a loading control.

To obtain populations of isotope-labeled tightly-synchronous cells progressing from G1 to S phase, we modified the Whitfield *et al.* (2002) double-thymidine block and release protocol (Materials and Methods) [7]. We released HeLa cells from the second thymidine block (“DT Block” = early S phase) to allow checkpoint recovery and normal passage through the subsequent transitions and allowed them to progress into mitosis without further chemical perturbation. We collected mitotic cells using a “shake-off” method, a procedure that takes advantage of the tenuous attachment of HeLa cells as they round up during mitosis. We replated mitotic cells in fresh dishes, and 3 hrs after mitosis, the cells were a relatively pure population of G1 cells; by 10 hrs after mitosis they were in early-S phase (Figure 1A and 1B show a full time course from cells grown in normal isotope medium). Note that these cell cycle times reflect a moderate delay compared to cells grown under standard conditions due to the requirement for dialyzed fetal bovine serum for efficient metabolic labeling.

To facilitate the detection of proteins that may be rapidly degraded in S phase we treated another culture of cells with the proteasome inhibitor MG132 8 hrs after the mitotic shake-off (just prior to the G1/S transition) and harvested the cells 2 hrs later in early S phase. To quantify proteins that change between S phase and G2 phase, we released cells into S phase from the double-thymidine block rather than from a mitotic shake-off. These cells progressed through S phase and entered G2 phase synchronously; we harvested 3 hrs (S phase) and 8 hrs (G2 phase) after release from the second thymidine block (Figure 1D and E show a full time course from cells grown in normal isotope medium). We also treated cells with MG132 6 hrs after release (just prior to the S/G2 transition) and harvested them 2 hrs later (G2 phase).

For the G1/S comparison, the G1 culture contained normal isotopes (light), the early-S phase culture was metabolically labeled with intermediate isotopes (medium), and the early-S phase culture treated with MG132 at the G1/S transition had been cultured in the heaviest isotopes (heavy). For the S/G2 comparison, mid-S phase cells were cultured in the normal isotope medium (light), the G2 cells were cultured in the intermediate isotope medium, and the G2 cells that had been treated with MG132 at the S/G2 transition were labeled in heavy isotope medium. In this manner, we generated synchronous metabolically-labeled cell populations naturally passing from one phase to the next without the potentially confounding issue of harvesting cells from a strong checkpoint arrest.

We confirmed cell cycle position by immunoblotting whole cell lysates for established cell cycle-regulated proteins. For example, we confirmed that both the Cdc6 and geminin proteins, two targets of the Anaphase Promoting Complex/Cyclosome (APC/C) E3 ubiquitin ligase which is active from anaphase through late G1, were substantially more abundant in the S phase lysates than in the G1 lysates (Figure 1C, compare lanes 2 and 3 to lane 1) [27], [28], [29], [30], [31]. In contrast to Cdc6 and geminin, the Cdt1 protein is targeted for degradation at the onset of S phase by the CRL4^Cdt2^ E3 ubiquitin ligase [32], [33]. As expected, we detected very little Cdt1 in the early-S phase cells compared to the G1 cells (Figure 1C, compare lanes 1 and 2), but Cdt1 protein levels were high in the S phase cells treated with MG132 (Figure 1C, compare lanes 2 and 3). Moreover, we observed higher levels of Cdt1 in the G2 samples compared to the mid-S phase samples as expected because CRL4^Cdt2^ can only target Cdt1 during active DNA replication (Figure 1F, compare lanes 1 and 2) [33], [34], [35].

Previously, we identified two proteins (SLBP and E2F1) that are degraded at the end of S phase as a result of Cyclin A/Cdk1 activation. Their degradation is blocked by MG132 treatment [36], [37], [38]. We detected not only the down-regulation of SLBP in G2 phase but also its stabilization in cells treated with MG132 (Figure 1F). Finally we confirmed that MG132 did not prevent S phase entry or exit as determined by flow cytometry and immunoblot analysis of marker proteins Figures 1A and 1D). We conclude therefore that these protocols generated synchronous populations that display the expected differences in protein abundance of known cell-cycle regulated proteins at the G1/S and S/G2 transitions.

### Protein Abundance Changes at the G1/S and S/G2 Transitions

**Figure 2:**
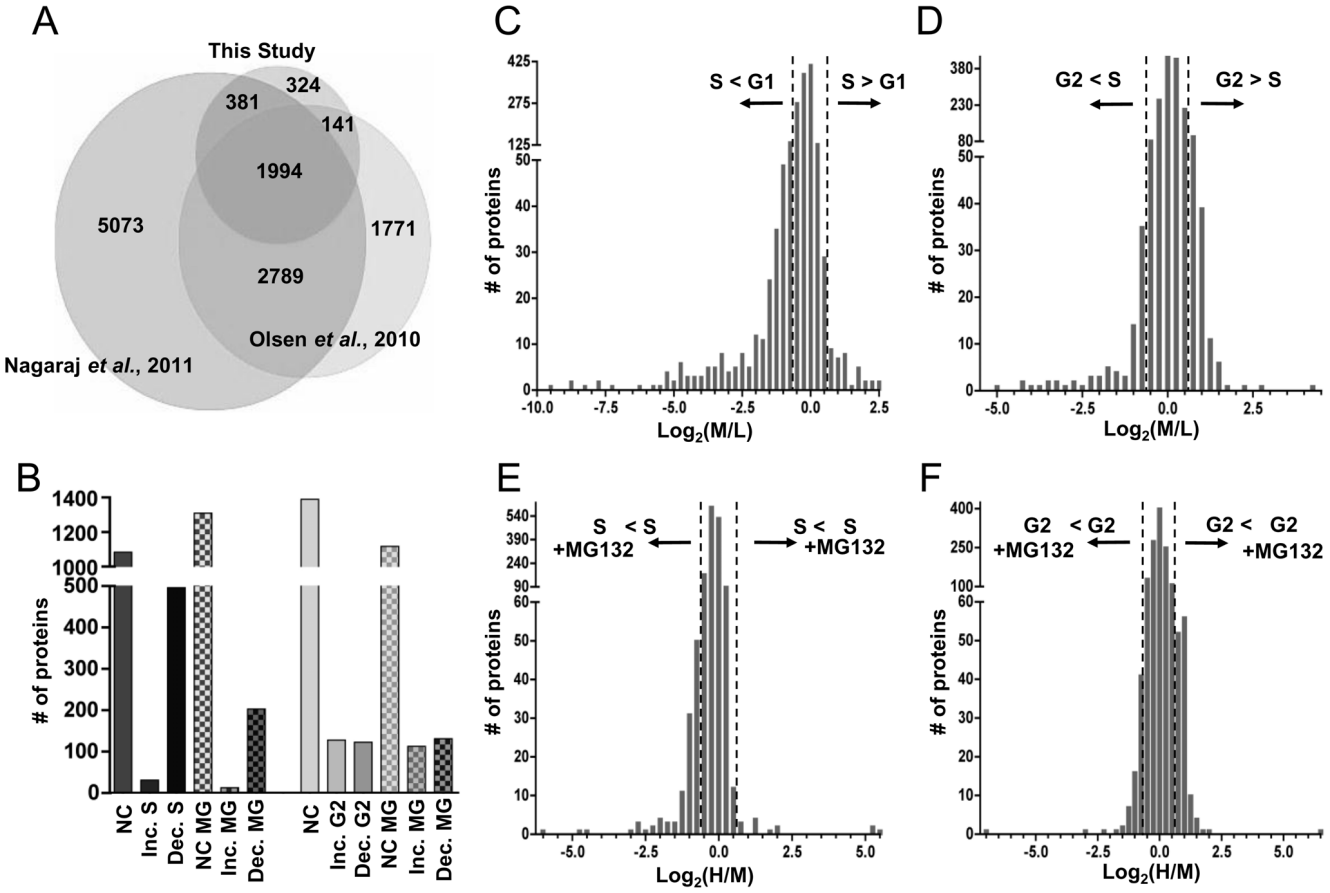
Cell cycle-regulated proteins from G1 to S and S to G2 detected by mass spectrometry. A) Comparison of the total number of proteins detected in this study (2,842 proteins) to two other studies of the HeLa cell proteome: Nagaraj et al., 2011 (10,237 proteins) [39] and Olsen et al., 2010 (6,695 proteins) [8]. B) Quantified proteins from this study were divided into lists based on their fold and direction of change; the total protein count for each list is plotted. “NC” denotes proteins that did not change. “NC MG,” “Inc MG,” and “Dec MG” denote proteins that either did not change, increased, or decreased in response to MG132 treatment, respectively. C) All quantifiable proteins in the G1 to S dataset plotted by their log_2_ transformed isotope ratios (medium S phase/light G1 phase). Dotted lines denote the 1.5-fold change threshold. D) All quantifiable proteins identified in the S to G2 dataset plotted by their log_2_ transformed isotope ratios (medium G2 phase/light S phase); dotted lines denote the 1.5-fold change threshold. E) Proteins identified in early-S phase cells compared to early-S phase cells treated with MG132 plotted by their log_2_ transformed isotope ratios (heavy S phase plus MG132/medium S phase minus MG132). Dotted lines denote the 1.5-fold change threshold. F) Proteins identified in G2 phase cells compared to G2 phase cells treated with MG132 plotted by their log_2_ transformed isotope ratios (heavy G2 plus MG132/medium G2 phase minus MG132). Dotted lines denote the 1.5-fold change threshold.

Using these validated samples from synchronous cells, we prepared whole cell lysates, combined the three lysates representing the G1/S comparison and the three lysates representing the S/G2 comparison, and subjected them to SDS-PAGE. We divided the gel into slices from which we generated tryptic peptides for liquid chromatography separation and tandem mass spectrometry (LC-MS/MS), as described in Materials and Methods. Using both MaxQuant and Proteome Discoverer software, we analyzed peptide spectra from a total of 50 gel slices. We identified 28,684 unique peptides corresponding to 2,842 unique proteins (allowable false discovery rate of 5%). Spectra were of sufficient quality to accurately quantify 2,410 of these proteins. A recent very comprehensive analysis of the HeLa proteome detected a total 10,237 proteins from lysates of asynchronous cells indicating that our analysis covers approximately 28% of the currently detectable HeLa proteome [39]. Note that quantitation requires detection of at least two isotopically labeled forms of the peptide, so any protein that was clearly detectable in only one of the three cultures was excluded from our analysis. Our dataset is also approximately 43% as extensive as another recent proteome analysis of HeLa cells that focused on changes during mitosis [8]. Interestingly, we detected 324 proteins not found in either previous report; these could reflect proteins that are only abundant enough for detection at specific cell cycle stages or could reflect random sampling differences among the three studies (Figure 2A). Therefore, our proteome analysis of the G1/S and S/G2 transitions complements and extends other investigations of the HeLa cell proteome.

To focus specifically on proteins that change in abundance from G1 to S phase, we compared the 1,611 quantifiable proteins (of 1,843 identified) from cells harvested in G1 to those from the subsequent early-S phase time point. We chose a 1.5-fold change in protein abundance as the threshold to score a protein as increased or decreased; these changes were calculated using the mean of all peptides from the same protein. Between these two cell cycle phases, two-thirds (67.3%) of the proteins neither increased nor decreased in abundance, whereas 32.7% either accumulated or decreased between G1 and S phase (Figure 2B and C). We quantified 1,640 proteins from the S/G2 comparison (of 1,913 identified). In contrast to the G1/S comparison, a higher proportion (84.7%) of these proteins did not change by more than 1.5-fold from S to G2 phase. Of the total quantifiable proteins, 15.3% either increased or decreased in their abundance (Figure 2B and D). These protein lists are provided in Tables S1 and S2, and the individual peptide lists are provided in Table S6.

The pharmacological inhibitor MG132 blocks the activity of the 26S proteasome, leading to the accumulation of proteins targeted for polyubiquitination [40], [41]. Since many cell cycle transitions are driven by ubiquitin-mediated protein degradation, we reasoned that we could identify some of these proteins based on altered abundance in the presence of MG132. It is important to note that MG132 was added close to the cell cycle transition under investigation. Overall, ∼1% of S phase proteins and 8% of G2 proteins were induced by MG132 treatment for 2 hrs compared to untreated early-S phase and G2 cells, respectively (Figure 2B, E and F, and Tables S3.1 and S4.1). We also detected proteins that were induced by treatment with MG132 that had not shown changes between cell cycle phases. These proteins could have short half-lives and be subject to continuous ubiquitin-mediated degradation at many or all cell cycle phases. Interestingly, more proteins were down-regulated after MG132 treatment than were induced - 13% of S phase and 10% of G2 proteins (Figure 2B, and Tables S3.2 and S4.2). A similar phenomenon has been reported previously; one study reported that 15% of proteins were down-regulated at least 2-fold after treating asynchronous cells with MG132 for 4 hrs [42]. The complete list of protein changes in response to MG132 treatment for both datasets is provided as Tables S3 and S4.

Some of the protein changes observed from one cell cycle phase to the next, such as cyclin B induction in G2, are well known. All the known cell cycle-regulated proteins that we detected changed as expected, although several relatively low abundance proteins were not detected. For example, the average abundance of peptides derived from ribonucleoside-diphosphate reductase subunit M2 (RRM2) increased 4.8-fold in S phase. This protein is regulated both at the transcriptional level, as a target of E2F4 repression, and at the protein level, as a target of the APC/C ubiquitin ligase [43], [44], [45].

**Figure 3:**
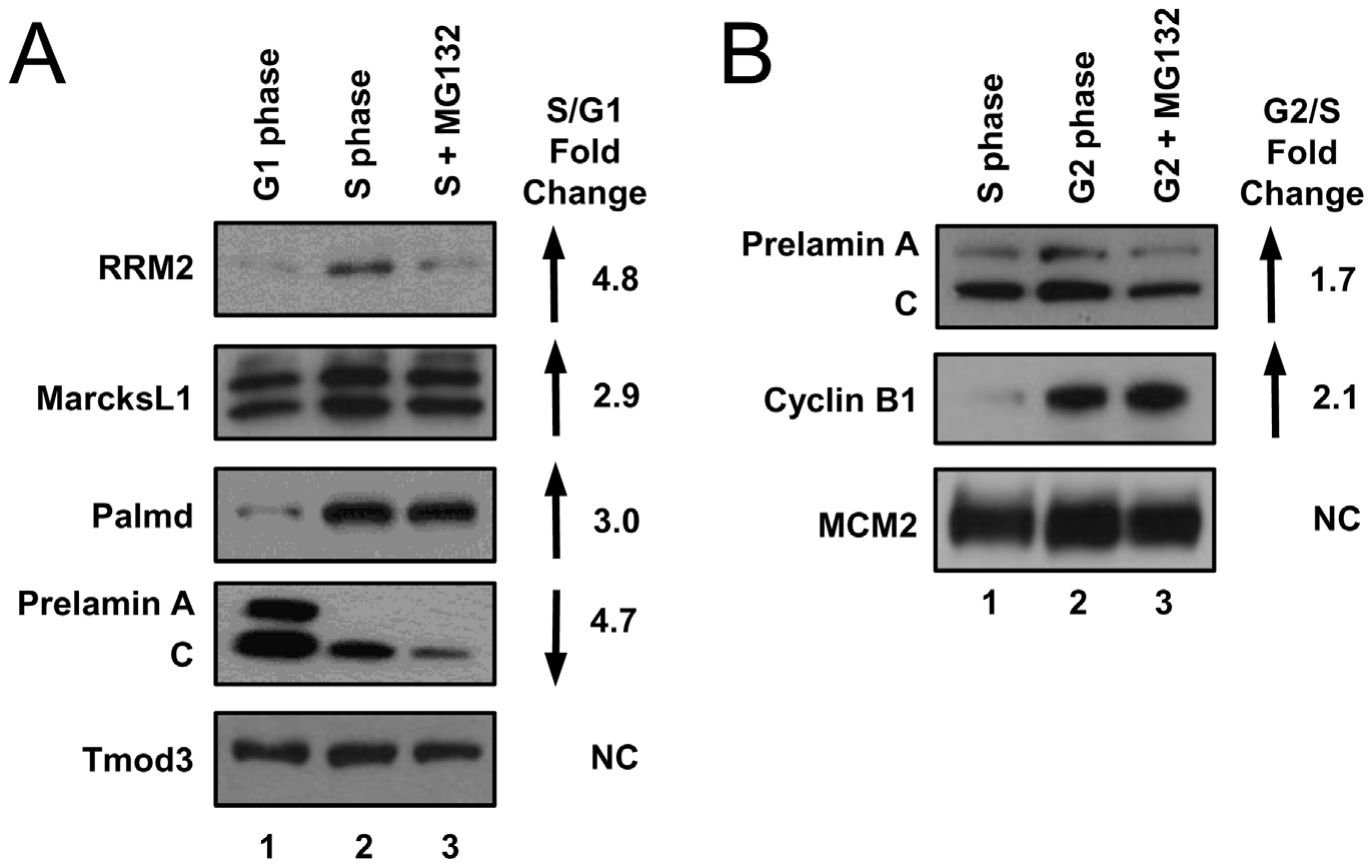
Validation of selected cell cycle-regulated protein predicted by mass spectrometry. The same cell lysates analyzed by mass spectrometry were subjected to immunoblot analysis for the indicated endogenous proteins in the A) G1 to S lysates or B) S to G2 lysates. Reported fold change ratios from mass spectrometry are listed to the right.

Our data also predicted changes in protein abundance that have not been previously identified. We selected several of these proteins for immunoblot validation on the original lysates of synchronized HeLa cells. Most of the proteins (17 out of 28) we selected for this validation showed changes in abundance that were consistent with the mass spectrometry quantification. For example, MARCKS-related protein (MARCKSL1) and palmdelphin (Palmd) increased in S phase compared to G1 phase by 2.9-fold and 2.0-fold, respectively, and we observed increases in band intensities for these proteins by immunoblotting (Figure 3A, compare lanes 1 and 2). Furthermore, mass spectrometry indicated that prelamin A/C protein levels decreased 4.7-fold in S phase compared to G1, and immunoblot analysis supported this finding (Figure 3A). As an example of a protein that does not change between G1 and S phase, we found that tropomodulin-3 (Tmod3) protein levels did not change significantly, in agreement with the mass spectrometry analysis. The total number of proteins that changed (increased or decreased) between S and G2 was smaller than the number of proteins that changed between G1 and S phase. We selected several proteins for validation by immunoblot analysis as above. For example, the average peptide abundance derived from prelamin A/C and cyclin B1 increased in G2 phase compared to mid-S phase by 1.7-fold and 2.1-fold, respectively; we observed changes in band intensities consistent with these mass spectrometry results (Figure 3B, compare lanes 1 and 2).

### Frequent Discordance of mRNA and Protein Abundance

**Figure 4:**
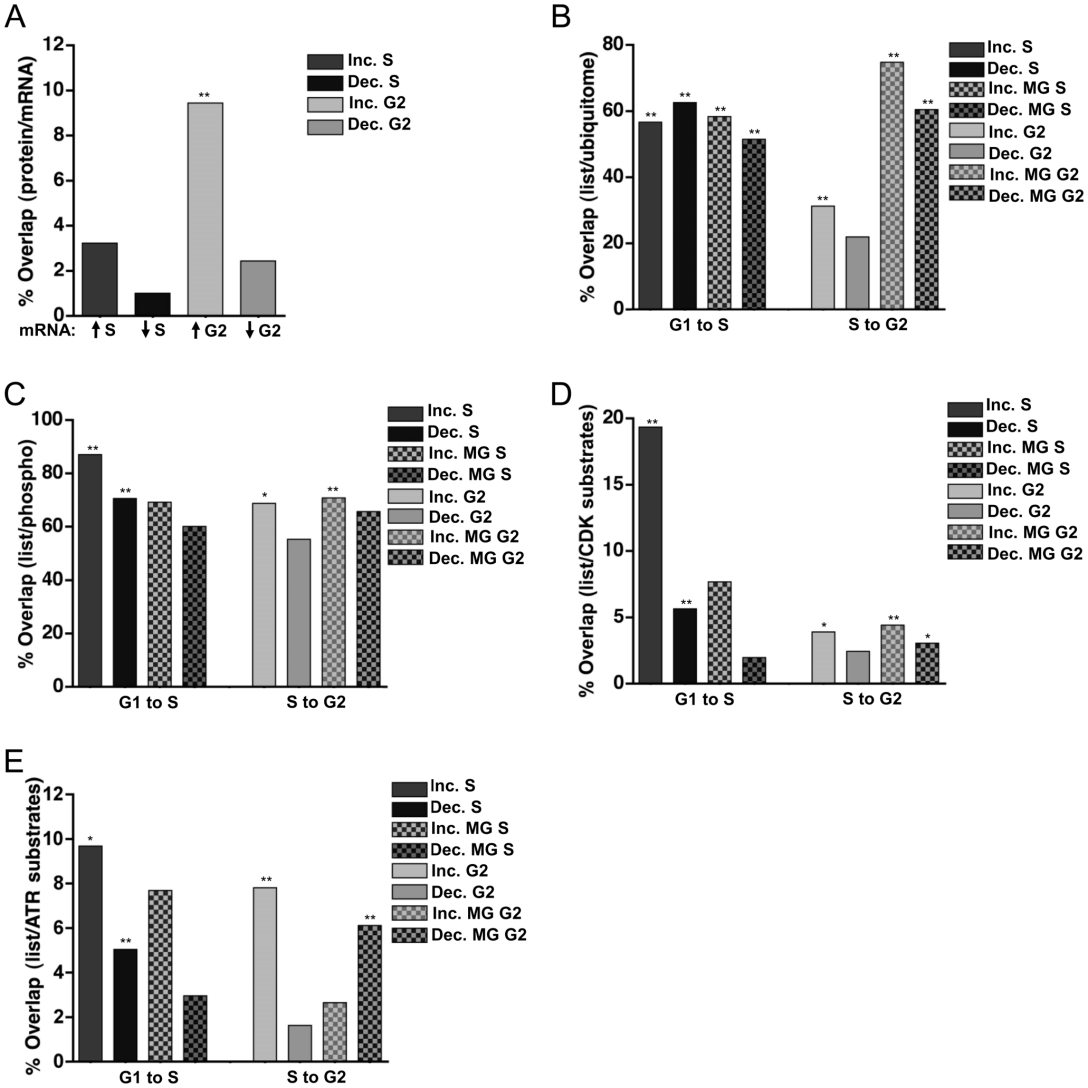
Discordance between mRNA and protein abundance. A) Individual lists of proteins that changed by at least 1.5-fold were compared to the mRNA data for those same proteins in synchronized HeLa cells from Whitfield et al. 2002 [7]. The percentage of proteins whose corresponding mRNA also changed is graphed for both S phase and G2 phase. ** p<0.001. B-E) Individual lists of proteins that changed by at least 1.5-fold were compared to proteins predicted to be B) ubiquitinated in asynchronous HCT116 cells [15], C) phosphorylated in HeLa cells [8], D) substrates of Cyclin A/Cdk2 [17], and E) substrates of the ATR kinase [16]. The percentage of each list that overlaps with the published dataset is plotted. * p<0.01; ** p<0.001.

Changes in protein abundance can often be explained by corresponding fluctuations in mRNA abundance. A landmark study by Whitfield et al. (2002) catalogued changes in mRNA expression through multiple synchronous cell cycles in HeLa cells [7]. The primary data from this extensive analysis is readily available for interrogation (cyclebase.org), and we sought to determine the relationship between mRNA expression in the Whitfield study with the protein changes we detected in this study. We divided the mRNA data into groups based on peak cell cycle phase of abundance [13], [14]. We then determined which of the proteins that changed from one cell cycle phase to the next in our study were also the products mRNAs whose abundance changed in the same way. Somewhat surprisingly, there was no significant overlap between the mRNAs that peak in S phase and the detected proteins that increased in S phase; likewise, proteins that decreased in S phase were unlikely to be the products of mRNAs that decreased in S phase (Figure 4A, first two bars). This poor correlation also existed when we compared proteins that increased in S phase to mRNAs that peaked in G1. As pointed out by Whitfield et al., there were fewer changes in mRNA levels between G1 and S phase than there were between S and M phase; only 19.5% of transcripts peak in S phase whereas 45% peak in G2/M [7].

In contrast, proteins that increased in G2 were somewhat more likely to be the products of mRNAs that also increased in G2 (Figure 4A, third bar). For example, the prelamin A/C mRNA peaks in G2/M, and the protein also modestly increased in our G2 samples compared to S phase (Figure 3B, compare lanes 1 and 2). In contrast, proteins that decreased in G2 were not well-predicted by mRNAs that also decreased in G2 (Figure 4A, fourth bar). Furthermore, when we compared the proteins that did *not* change in either of our datasets to the mRNAs that are constitutively expressed throughout the cell cycle, more than 60% of the genes/proteins were in agreement (Figure S1, first two bars). When the set of constitutive proteins were compared to the mRNAs that fluctuate, this overlap was much smaller, though still statistically significant (Figure S1). Thus, some of the proteins whose abundance did not change by mass spectrometry analysis are the products of mRNAs that do change; these proteins may be long-lived and thus not fully reflective of corresponding mRNA changes.

Since mRNA abundance could not fully account for the protein changes we observed, we considered the possibility that the changes in protein abundance were correlated with ubiquitination and thus, regulated protein degradation. We compared our lists of proteins that change from G1 to S or from S to G2 to a recently-published list of ubiquitinated proteins identified in asynchronously growing HCT116 (human colon carcinoma) cells [15]. Strikingly, a high proportion of the proteins that either increased (56.7%) or decreased (62.6%) between G1 and S also appeared in the list of 4,462 ubiquitinated proteins (Figure 4B, first two bars). Moreover, proteins whose abundance was affected by MG132 treatment in S phase (either increased or decreased) were also highly represented in the reported list of total ubiquitinated proteins. In contrast, proteins that changed from S to G2 were not as enriched in the “ubiquitome,” regardless of MG132 treatment with the exception of proteins that increased from S phase to G2 (Figure 4B).

Both nuclear and cytoplasmic proteins were present in all of our datasets, and we detected no differences in nuclear-cytoplasmic localization among proteins that changed from one cell cycle phase to the next (Figures S2A and S2B). A strikingly large proportion of proteins whose abundance changed from G1 to S or from S to G2 have been detected as phosphoproteins, consistent with the notion that many protein abundance changes are controlled by phosphorylation (Figure 4C). This enrichment was true both for proteins that changed from G1 to S and for those that changed from S to G2.

Since the cyclin-dependent kinases (Cdks) govern many cell cycle transitions, we compared our sets of regulated proteins with a list of candidate Cdk substrates [17]. Many proteins that increased (6 of 31) or decreased (28 of 496) in S phase appear on this list of Cdk substrates (Figure 4D, first two bars). Moreover, a statistically significant number of proteins that increased in G2 phase are also putative Cdk substrates (Figure 4D, fifth bar). A significant number of proteins that changed with MG132 treatment at the S/G2 transition are also putative Cdk substrates (Figure 4D, last two bars). In contrast, proteins that changed in response to MG132 treatment at the G1/S transition were not enriched for putative Cdk substrates (Figure 4D, third and fourth bars).

Like Cdks, the ATR kinase is active during S phase [46]. ATR activity is also stimulated by DNA damage, and this property was used to identify candidate ATR substrates. Putative ATR kinase substrate lists were developed by Stokes et al. (2007) from phosphopeptides detected following UV irradiation, an activator of ATR [16]. A subset of our regulated proteins also appeared in these lists of potential ATR substrates (Figure 4E). The majority of proteins that change with MG132 treatment, (both lists), were not ATR substrates, but proteins that decreased with MG132 treatment at the S/G2 transition were significantly enriched in ATR substrates (Figure 4E). Taken together, these comparisons are consistent with the prevailing model that many changes in protein abundance between G1 and S phase and between S and G2 phase are associated with both protein ubiquitination and protein phosphorylation, but this analysis also underscores the idea that only some changes, particularly as cells progress from G1 to S phase in continuously growing cells, are due solely to mRNA fluctuations.

### Unanticipated Cell Cycle-regulated Proteins Include Alternative pre-mRNA Splicing Factors

To determine which biological processes might be cell cycle-regulated, we analyzed the Gene Ontology (GO) enrichment of each of our lists. As expected, “cell cycle” was enriched in our sets of cell cycle-regulated proteins (increase in G2). The three most highly-enriched terms for each list are shown in Table 1, and the full list is provided in Table S5. Proteins involved in cell morphogenesis increased from G1 to S phase, whereas proteins assigned to the GO term “protein folding” decreased (Table 1) from S to G2 phase. Surprisingly, proteins involved in RNA processing and ribonucleoprotein complex biogenesis were significantly represented in the set of proteins that decreased from G1 to S phase and the set that increased from S to G2 phase. (The proteins that decreased from G1 to S phase are not necessarily the same proteins that were increased in the S to G2 dataset.) Both sets of MG132-sensitive proteins were also enriched for RNA processing and ribonucleoprotein complex biogenesis proteins (Table 2).

**Table 1:** Top three significant GO terms enriched in individual lists of cell cycle-regulated proteins.

Increase in S phase		
GO Term	p-value	Protein Count
Regulation of cell morphogenesis	0.001	4
Negative regulation of cellular component organization	0.024	3
Negative regulation of cell projection organization	0.047	2
**Decrease in S phase**		
**GO Term**	**p-value**	**Protein Count**
RNA processing	3.96e−34	83
Ribonucleoprotein complex biogenesis	1.98e−20	38
Translational elongation	2.46e−18	28
**Increase in G2 phase**		
**GO Term**	**p-value**	**Protein Count**
RNA processing	2.25e−05	16
Cell cycle	0.001	16
Cellular protein localization	0.002	11
**Decrease in G2 phase**		
**GO Term**	**p-value**	**Protein Count**
Protein folding	0.007	6
Macromolecular complex assembly	0.015	11
Positive regulation of anti-apoptosis	0.018	3

**Table 2:** Top three significant GO terms enriched in the individual lists of MG132-sensitive proteins.

Increase in S phase following MG132 treatment		
GO Term	p-value	Protein Count
Signal complex assembly	0.009	2
Cell migration	0.011	3
Cellular macromolecular complex assembly	0.014	3
**Decrease in S phase following MG132 treatment**		
**GO Term**	**p-value**	**Protein Count**
Ribonucleoprotein complex biogenesis	2.59e−10	17
Ribosome biogenesis	1.57e−07	12
RNA processing	3.09e−07	23
**Increase in G2 phase following MG132 treatment**		
**GO Term**	**p-value**	**Protein Count**
Translational elongation	5.44e−130	68
Ribosome biogenesis	1.01e−14	16
Ribonucleoprotein complex biogenesis	2.13e−13	17
**Decrease in G2 phase following MG132 treatment**		
**GO Term**	**p-value**	**Protein Count**
Protein transport	1.45e−05	20
Protein localization	3.30e−05	21
mRNA processing	7.03e−05	12

**Figure 5:**
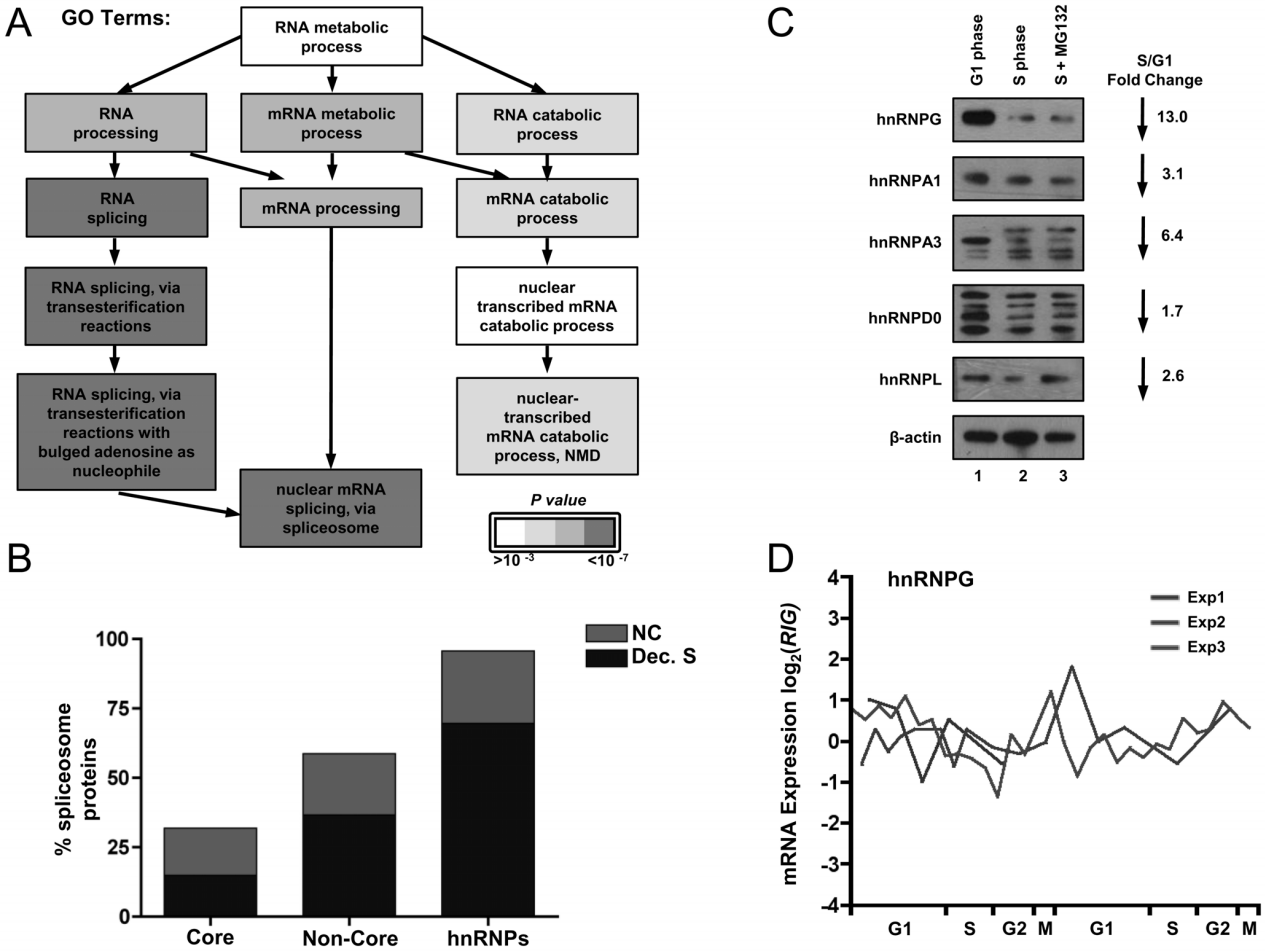
pre-mRNA alternative splicing factors are enriched among proteins that decrease from G1 to S phase. A) The GO term analysis tree of a branch of RNA metabolism is shaded to indicate decreasing p-values for the enrichment in the protein datasets of this study. B) Spliceosome proteins were designated as either core or non-core proteins; hnRNPs represent a subset of the non-core spliceosome proteins [47]. The total percentage of the category of splicing proteins is plotted. The portion of the bars shaded blue represents the percentage that decreased between G1 phase and S phase, and the portion shaded green represents the fraction that did not change between G1 and S phase. The full list of splicing proteins quantified is provided in Table S7. C) Whole cell lysates from synchronized cultures (Figure 1C) were analyzed for the indicated endogenous hnRNP proteins; the fold change ratios from mass spectrometry are listed to the right. β-actin serves as a loading control. D) mRNA abundance for the hnRNPG gene was extracted from the Whitfield *et al.* (2002) dataset [7]; expression data from 3 double-thymidine block and release experiments are shown as a function of cell cycle phase.

The striking enrichment of pre-mRNA processing proteins in the collection of proteins that were down-regulated in S phase prompted us to analyze those proteins more directly. In particular, the enriched GO terms included nuclear pre-mRNA splicing, and more specifically, alternative splicing (Figure 5A). Of the 244 known splicing factors, we detected 72 core proteins and 65 non-core proteins (Table S7) [47]. Overall, we detected 31.9% of the core spliceosome proteins, of which 46.7% decreased in S phase (Figure 5B, first bar). Of note, proteins in the U2 complex decreased, suggesting that a specific part of the core machinery may be regulated during S phase. Additionally, we detected 58.7% of the non-core spliceosome machinery, and 62.3% of these subunits decreased in S phase (Figure 5B, second bar). Strikingly, we quantified almost all (95.7%) of the known heterogeneous nuclear ribonucleoproteins (hnRNPs), and 72.7% of these proteins decrease in S phase (Figure 5B, third bar). These proteins are important in determining exon inclusion, suggesting that alternative splicing is particularly affected during S phase [48], [49], [50], [51].

We probed several of the alternative splicing factors by immunoblotting to determine if the changes observed by mass spectrometry were valid. As shown in Figure 5C, several hnRNPs decreased between G1 and S phase, such as hnRNPG, hnRNPA1, and hnRNPL (compare lanes 1 and 2). For two other proteins, hnRNPA3 and hnRNPD0, we detected multiple isoforms that clearly changed between G1 and S phase. Some isoforms decreased in abundance but new isoforms accumulated in the S phase samples (Figure 5C, compare lane 1 with lanes 2 and 3). Of note, the hnRNPA3 protein has been reported to be heavily phosphorylated, raising the possibility that the decrease observed by mass spectrometry was due to cell cycle regulated post-translational modifications [52], [53], [54], [55], [56], [57], [58], [59]. Indeed, a number of hnRNPs, including hnRNPD0, were identified as Cyclin A/Cdk2 substrates [17]. Moreover, we confirmed S phase downregulation of hnRNPG in biological replicates of synchronized HeLa cells (Figure S3A) and S phase downregulation of hnRNPA3 in another line, T98G (Figure S3B). Additionally, none of the splicing proteins that decreased in S phase were the products of mRNAs that also decreased in S phase (for example, hnRNPG is shown in Figure 5D, others in Figure S4), suggesting that their regulation must be posttranscriptional.

## Discussion

Previous unbiased analyses of the human transcriptome and proteome have generated an appreciation for the interconnectedness of different biochemical pathways. Inspired by such findings, we considered it likely that the human cell cycle includes changes not only in the well-studied processes of chromosome replication, mitosis, and cell division, but also changes in other cellular processes. This hypothesis was supported by our discovery that proteins involved in alternative pre-mRNA splicing are down-regulated in S phase. The reason for this apparent systemic regulation of pre-mRNA splicing has yet to be elucidated, but could reflect a need to rapidly alter the isoforms of a cohort of proteins from one cell cycle phase to the next. The depth of our proteome coverage likely reflects changes in the most abundant and readily detectable proteins; thus these fluctuations indicate novel biological pathways and processes that are cell cycle-regulated even when the rarest proteins were not quantified.

Alternative splicing, particularly the production of different isoforms of specific mRNAs at different times in the same cell, is determined by *cis* elements (splicing enhancers and splicing silencers) and the relative concentrations of the *trans* factors, splicing activators and repressors (reviewed in ref. [60]). Changes in the relative concentrations of these regulatory proteins are responsible for most of the changes observed in alternative splicing. Thus, relatively small changes in the concentrations of these common splicing regulatory proteins, particularly the hnRNPs and SR proteins, can result in changes in a number of coordinately regulated alternative splicing events [61], [62], [63], [64].

This study extends and complements the cell cycle proteome analysis by Olsen et al. [8]. Our cells were not only very tightly synchronized in early S phase by the double-thymidine and mitotic shakeoff protocol, but importantly, we collected cells as they progressed synchronously through the cell cycle *after* release from the block. This protocol is distinct from other popular synchronization methods in which cells were harvested while chemically arrested with replication or mitotic inhibitors or were harvested very shortly after release from such inhibitors. Likely due to these differences, a comparison of proteins that change from G1 to S or from S to G2 in our dataset to those reported by Olsen et al. (using a single block and release or nocodazole block and release) showed little overlap. Nevertheless, the alternative splicing factors we detected were also reported in the Olsen dataset, although the amplitudes of those changes were less than those we measured. These differences may be due to technical variations in culture conditions (for example, adherent vs. suspension cultures) or to differences in the degree of cell cycle synchrony. One area of close agreement between the two studies, however, is the conclusion that only a subset of cell cycle-regulated changes in protein abundance can be accounted for by changes in mRNA abundance.

Although many protein changes detected in this study did not match corresponding changes in mRNA levels, we noted a clear difference between the degree of concordance of the mRNA changes and protein changes between the two G1-to-S and S-to-G2 datasets. Proteins that increased from S to G2 were more likely to be the products of mRNAs that showed similar cell cycle-dependent changes, though these mRNA changes were only able to predict ∼10% of these G2-inducible proteins (Figure 4A). This relationship is consistent with the finding that 45% of the cell cycle regulated mRNAs peak in G2/M [7]. Strikingly, more than half of the proteins that changed – either increased or decreased – from G1 to S phase are among those reported to be polyubiquitinated, but this enrichment was much less or non-significant for proteins that changed from S to G2 (Figure 4B). Taken together, our analysis is consistent with the notion that protein changes from S to G2 are somewhat reflective of changes in mRNA levels, but proteins that change from G1 to S are reflective of ubiquitin-mediated protein degradation and phosphorylation.

Given the importance of ubiquitin-mediated protein degradation in cell cycle transitions, and that a number of cell cycle regulators change concentrations rapidly without concomitant changes in mRNA concentrations, we included analysis of cells treated with the proteasome inhibitor MG132. A relatively small number of proteins that increase after MG132 treatment at the G1/S transition were detected, whereas a larger number of MG132-inducible proteins were detected in cells treated at the S/G2 transition (Figure 2B and Tables S3.1 and S4.1). Interestingly, at least as many proteins were MG132-*repressible* as were MG132-inducible in both experiments (Figure 2B and Tables S3.2 and S4.2). Given the mechanism of action of MG132 as a competitive inhibitor of the 26S proteasome, we interpret these changes as a reflection of indirect cellular responses to the accumulation of polyubiquitinated proteins or the prevention of degradation of specific proteins. Some of the MG132-repressible proteins may themselves be targets for negative regulation by MG132-inducible repressors. Those targets of negative regulation would therefore be indirectly repressed by MG132. In addition, the loss of proteasome function may trigger a cellular stress response that is reflected in the proteome as down-regulation of a cohort of proteins. Of note, proteasome inhibitors are a chemotherapeutic strategy for anti-cancer treatment [65], [66], and prolonged treatment of HeLa cells with MG132 (e.g. 24 hrs) results in apoptosis [67]. Our report here of proteins whose levels change in response to MG132 at two specific cell cycle phases sheds additional light on the biological responses to such strategies.

A major challenge in this type of study is the detection of relatively low abundance proteins, many of which are critical regulators of cellular processes. Many of the previously defined cell cycle regulated proteins, often regulated by proteolysis, were not detected. These include SLBP, a critical regulator of histone mRNA metabolism, the E2F1-3 transcription factors, which are essential for the transcription of S phase genes, and many proteins needed for the formation of the pre-replication complex (Orc subunits, Cdc6, Cdt1, etc.). Detection of these low abundance proteins will require further advances in proteomics technology, perhaps through some method that removes the most abundant proteins, similar to how “ribo-minus” technology removes the most abundant RNAs to allow the detection of very low abundance RNAs by high-throughput sequencing.

Studies such as the one presented here add to our general knowledge of the global changes that can occur during the cell cycle. We expect that the combination of this analysis with other studies focused on mitosis, the phosphoproteome, the transcriptome, the ubiquitome, cell cycle changes in model organisms, etc. will facilitate a complete systems-level understanding of the cell cycle.

## Supporting Information

Figure S1 **Proteins that did not change in either the G1 to S or the S to G2 dataset were compared to mRNAs that were ubiquitously expressed or peaked at the indicated cell cycle phases** [**7**] **.** * p<0.01; ** p<0.001.
(PDF)

Figure S2 **Individual lists were compared to the Boisvert et al. (2012) data, which examined the subcellular location of proteins** [**18**] **.** “Ubiquitous” denotes proteins that were found in both the nuclear and cytoplasmic fractions, whereas “Nuclear” or “Cytoplasmic” proteins were found only in that compartment. Data from the A) G1 to S dataset and B) the S to G2 dataset are represented as the percentage of the individual list that overlaps with the published dataset. * p<0.01; ** p<0.001.
(PDF)

Figure S3 A) HeLa cells were synchronized as in Figure 1A and the endogenous levels of hnRNPG were examined. A non-specific band (NSB) was used as a loading control. B) T98G cells were synchronized in quiescence by serum starvation and stimulated to re-enter the cell cycle with 10% FBS; S phase entry begins at 20 hr. post-serum addition [9]. Lysates were analyzed for levels of endogenous hnRNPA3; α-tubulin serves as a loading control.
(PDF)

Figure S4 **Individual mRNA abundance data were extracted from the Whitfield et al. (2002) dataset** [**7**] **; expression data from 3 double-thymidine block and release experiments are shown as a function of cell cycle phase for A) hnRNPA1, B) hnRNPA2/B1, C) hnRNPD, and D) hnRNPL.**
(PDF)

Table S1 **Combined protein IDs and quantitation ratios for the G1 to S dataset.**
(XLS)

Table S2 **Combined protein IDs and quantitation ratios for the S to G2 dataset.**
(XLS)

Table S3 **Protein changes induced by MG132 added at the G1/S phase transition and harvested 2 hrs later in early S phase.**
(XLS)

Table S4 **Protein changes induced by MG132 treatment at the S/G2 transition and harvested 2 hrs later in G2 phase.**
(XLS)

Table S5 **Full GO term analysis of individual protein lists.**
(XLS)

Table S6 **Peptide IDs and quantitation ratios for both datasets.**
(XLS)

Table S7 **Splicing proteins down-regulated in S phase.**
(XLS)
