# Supplementary figures and images for: Cell Cycle-Regulated Protein Abundance Changes in Synchronously Proliferating HeLa Cells Include Regulation of Pre-mRNA Splicing Proteins
Source: PLoS One. 2013 Mar 8;8(3):e58456. doi: 10.1371/journal.pone.0058456 (PMC3592840; doi:10.1371/journal.pone.0058456)

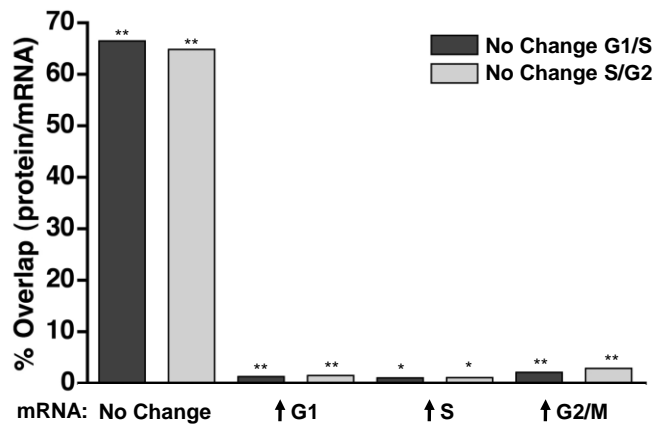

Supplement: Figure S1 — Proteins that did not change in either the G1 to S or the S to G2 dataset were compared to mRNAs that were ubiquitously expressed or peaked at the indicated cell cycle phases [7] . * p<0.01; ** p<0.001. (PDF) [file pone.0058456.s001.pdf]

A

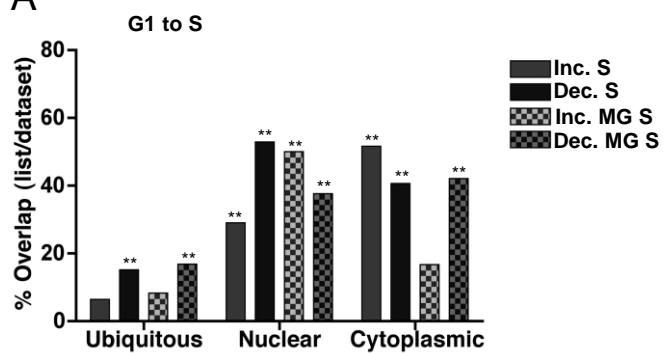

B

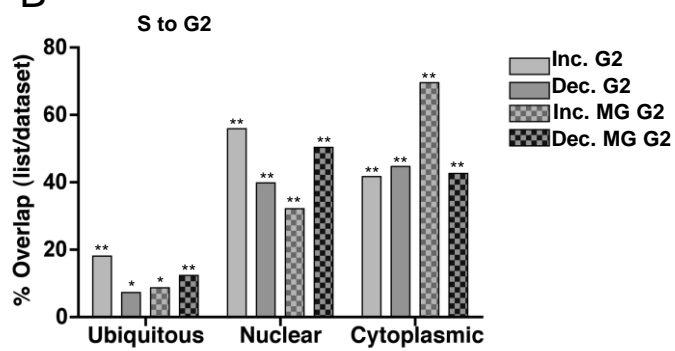

Supplement: Figure S2 — Individual lists were compared to the Boisvert et al. (2012) data, which examined the subcellular location of proteins [18] . “Ubiquitous” denotes proteins that were found in both the nuclear and cytoplasmic fractions, whereas “Nuclear” or “Cytoplasmic” proteins were found only in that compartment. Data from the A) G1 to S dataset and B) the S to G2 dataset are represented as the percentage of the individual list that overlaps with the published dataset. * p<0.01; ** p<0.001. (PDF) [file pone.0058456.s002.pdf]

A

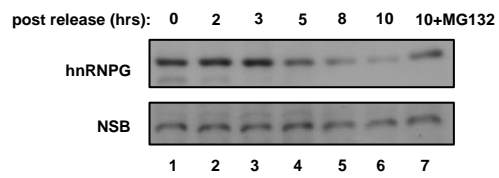

B

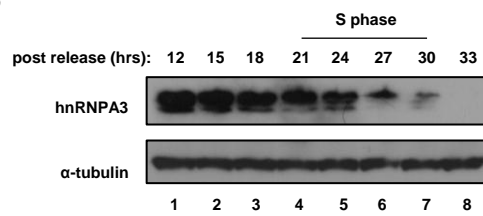

Supplement: Figure S3 — A) HeLa cells were synchronized as in Figure 1A and the endogenous levels of hnRNPG were examined. A non-specific band (NSB) was used as a loading control. B) T98G cells were synchronized in quiescence by serum starvation and stimulated to re-enter the cell cycle with 10% FBS; S phase entry begins at 20 hr. post-serum addition [9]. Lysates were analyzed for levels of endogenous hnRNPA3; α-tubulin serves as a loading control. (PDF) [file pone.0058456.s003.pdf]

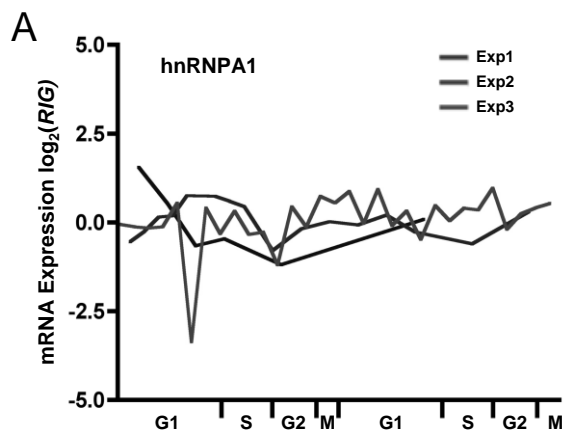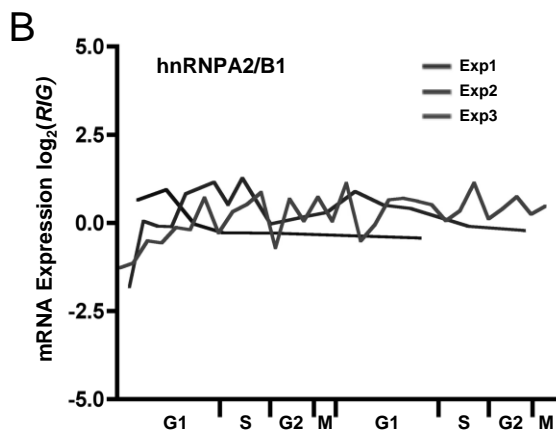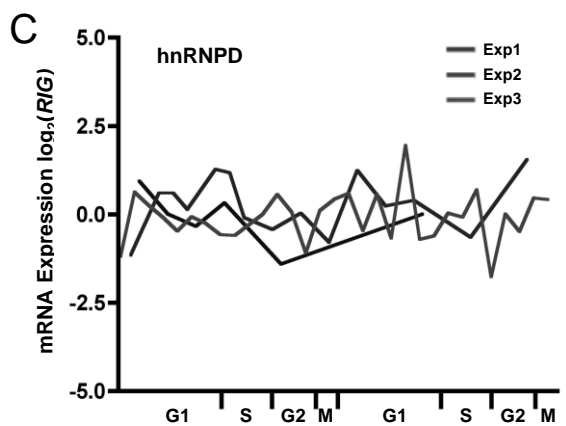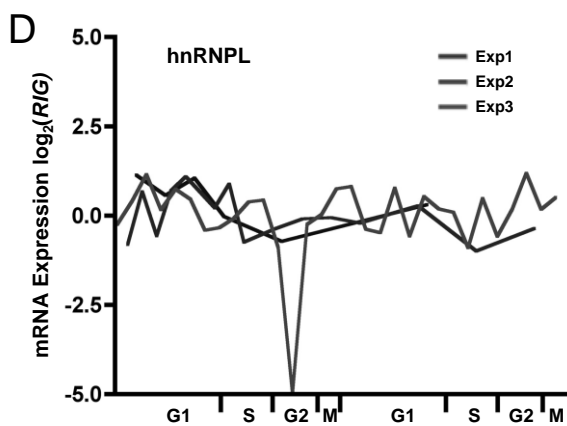

Supplement: Figure S4 — Individual mRNA abundance data were extracted from the Whitfield et al. (2002) dataset [7] ; expression data from 3 double-thymidine block and release experiments are shown as a function of cell cycle phase for A) hnRNPA1, B) hnRNPA2/B1, C) hnRNPD, and D) hnRNPL. (PDF) [file pone.0058456.s004.pdf]
